# Real-Time Imaging of Single Retinal Cell Apoptosis in a Non-Human Primate Ocular Hypertension Model

**DOI:** 10.1167/tvst.13.1.20

**Published:** 2024-01-22

**Authors:** Takeshi Ishikawa, Naoki Kishi, Yoshiko Shimizu, Takao Fujimura, Takao Yamazaki

**Affiliations:** 1 Translational Science Management, Non-Clinical Biomedical Science, Astellas Pharma Inc., Tsukuba, Japan; 2 Portfolio Evaluation Group, Cooperate Strategy, Astellas Pharma Inc., Tokyo, Japan; 3 Product Creation Unit, Immuno-Oncology, Astellas Pharma Inc., Tsukuba, Japan

**Keywords:** apoptosis, retinal imaging, experimental glaucoma, biomarkers

## Abstract

**Purpose:**

To evaluate the feasibility of using DARC (detection of apoptosing retinal cells) technology as a biomarker for preclinical assessment of glaucomatous damage in a non-human primate (NHP) model of ocular hypertension (OHT).

**Methods:**

Elevated intraocular pressure (IOP) was induced by applying a laser to the trabecular meshwork in each eye of NHPs. Changes in DARC counts in the retina, identified as fluorescent-tagged annexin V (ANX776)–positive cells, were evaluated together with optic nerve damage, assessed using spectral domain-optical coherence tomography. The pharmacokinetic properties of ANX776 in both healthy and OHT model monkeys were also examined.

**Results:**

Sustained elevation of IOP and subsequent thinning of the retinal nerve fiber layer thickness (RNFLT) around the optic nerve head were confirmed in the OHT model. Increases in DARC counts were also detected after IOP elevation. We identified a statistically significant relationship between cumulative DARC counts and reductions in RNFLT both globally and in each peripapillary sector. Intravenous administration of ANX776 increased blood annexin V in a dose-dependent manner, which was subsequently eliminated.

**Conclusions:**

This study revealed that DARC technology can effectively assess glaucomatous damage in an NHP OHT model. We obtained the fundamental data that could serve as a reference for developing preclinical models to evaluate the pharmacodynamics of neuroprotective agents using DARC technology in NHP OHT models.

**Translational Relevance:**

Our basic data in a monkey OHT model could be useful for future preclinical studies using DARC technology to estimate the pharmacodynamic response of neuroprotective agents.

## Introduction

Glaucoma is a leading cause of irreversible blindness worldwide^1^ and is characterized by degeneration and loss of retinal ganglion cells (RGCs) and their axons via apoptosis.^2^^,^^3^ Although elevated intraocular pressure (IOP) is currently the only modifiable risk factor, a proportion of glaucoma patients continue to lose their vision despite effective IOP control. Therefore IOP-independent risk factors are increasingly thought to play a role in the pathology of glaucoma, and targeting these factors may lead to potential new treatments for glaucoma. Neuroprotection has gained substantial interest in recent years as a therapeutic approach for preventing neuronal degeneration and loss of visual function in glaucoma.

Gaasterland and Kupfer^4^ first established a laser-induced chronic intraocular pressure (IOP) model of glaucoma in nonhuman primates (NHP) in 1974. The model is still used in ocular research with a reproducible IOP which shows a plateau in IOP for up to 10 weeks (70 days).^5^^,^^6^ The model is attractive because the nature of the insult (i.e. IOP elevation due to reduced outflow through the iridocorneal angle) resembles the probable mechanism of elevated IOP in humans with primary open angle glaucoma (POAG). Additionally, the anatomy of the optic nerve head (ONH) and macula, as well as the visual system in NHPs is very similar to that in humans. The laser-induced NHP model of ocular hypertension (OHT) is therefore thought to allow for greater translatability than small animal models of glaucoma. With these advantages, the NHP OHT model has been used to assess many medical treatments for glaucoma.

Detection of apoptosing retinal cells (DARC) is a retinal imaging technology that uses fluorescent-tagged annexin V (ANX776),^7^ which binds to exposed phosphatidylserine, to identify stressed and apoptotic retinal cells in the living eye.^8^ DARC has been used to evaluate the neuroprotective effects and treatment efficacy of an investigational drug in preclinical settings.^9^ In glaucoma clinical trials, DARC has been shown to predict patients at risk of disease progression 18 months before OCT changes arise.^10^

Here, we examined the feasibility of using DARC as a biomarker of glaucoma in the laser-induced NHP OHT model and compared the results of DARC with changes in peripapillary retinal nerve fiber layer thickness (RNFLT), an RGC-related parameter.

## Methods

### Experimental Animals

Experiments were performed at Shin Nippon Biomedical Laboratories (SNBL; Kagoshima, Japan) on eight male cynomolgus monkeys (Macaca fascicularis) aged 2 to 5 years at initiation of the study. Animals were kept under artificial light for 12 hr/day (7:00 AM to 7:00 PM) and were allowed free access to water and given food once a day. All animals were acclimated to the experimental environment for at least 1 week before use. All animal experimental procedures were approved by the Institutional Animal Care and Use Committee of Astellas Pharma Inc (approval no. C-T21077 for the pharmacokinetics [PK] study using healthy monkeys and C-T21221 for study using OHT model monkeys). Furthermore, all animal experimental procedures were approved by the Institutional Animal Care and Use Committee of SNBL (approval no. IACUC521-002 for PK study using healthy monkeys and IACUC501-290 for study using OHT model monkeys) and conformed to the ARVO statement on the Use of Animals in Ophthalmic and Vision Research. This study was performed in accordance with the animal welfare bylaws of SNBL, which is accredited by AAALAC International. Efforts were made in all procedures to minimize potential suffering of the animals.

### Establishment of the OHT Model

Four cynomolgus monkeys (male; body weight, 2.48 to 3.00 kg; age 2 years at initiation of the study) were used to develop the OHT model at SNBL, as described previously.^11^ Briefly, animals were anesthetized by intramuscular injection (0.2 mL/kg) of ketamine (Ketalar for Intramuscular Injection 500 mg; Daiichi Sankyo Propharma Co., Ltd., Tokyo, Japan, 50 mg/mL) and xylazine (Selactar 2% Injection Solution; Bayer Japan, Osaka, Japan, 20 mg/mL) mixture solution in a 7:1 (v/v) ratio. An ocular single mirror gonio lens (Ocular Instruments, Bellevue, WA, USA) was placed directly on the cornea along with hydroxyethyl cellulose (Scopisol solution for eye; Senju Pharmaceutical, Osaka, Japan). Green laser photocoagulation burns were applied at a wavelength of 532 nm for uniform 360° irradiation around the trabecular meshwork in both eyes of all monkeys. A 532-nm green laser (power, 1000 mW; spot size, 100 µm; duration, 0.2 second) was applied using a multicolor scan laser photocoagulator (MC-500; Nidek Co., Aichi, Japan). After the laser application, levofloxacin hydrate (Cravit ophthalmic solution 0.5%, Santen Pharmaceutical Co, Osaka, Japan) was instilled into the eyes once daily for 3 days from the day after application. In animals with an IOP below 30 mm Hg, additional laser treatment was applied to the relevant eye 14 days after the first treatment.

### IOP Measurement

IOP measurement was conducted using a tonometer (Icare TONOVET tonometer TV01; Icare Finland Oy, Vantaa, Finland). In eyes that received single laser application, IOP measurements were conducted before (pre = 0) and 1, 2, 4, 6, 8, and 10 weeks after the first laser application. In eyes that received double laser application, IOP measurements were conducted before the first laser application (pre = 0), 1 and 2 weeks after the first laser application (excluded from analysis because of the unchanged IOP) and 1, 2, 4, 6, 8, and 10 weeks after the second laser application. At each time point, IOP (mm Hg) was determined bilaterally 3 times in conscious animals restrained using a custom-made restrainer. The median value of the 3 measurements was adopted as the reported IOP.

### ANX776 Administration, SLO Imaging, and DARC-Positive Cell Counting

To acquire images for DARC counting, animals were given an ocular instillation of a mydriatic (Mydrin-P; Santen, Osaka, Japan), anesthetized with a 9:1 (v/v) mixture solution of ketamine and xylazine by intramuscular injection (0.2 mL/kg) and then subject to scanning laser ophthalmoscope (SLO) imaging. In animals that received single laser application in both eyes, administration of ANX776 and subsequent SLO imaging was conducted before (pre = 0) and 6, 8, and 10 weeks after the first laser application. In animals that received double laser application in both eyes, ANX776 administration and subsequent SLO imaging were conducted before the first laser application (pre = 0) and 6, 8, and 10 weeks after the second laser application. In animals that received single laser application in one eye and that received double laser application in the other eye, administration of ANX776 was conducted before (pre = 0), and 6, 8, 10, and 12 weeks after the first laser application (that is, 4, 6, 8, and 10 weeks after the second laser application), and SLO imaging was conducted before the first laser application (pre = 0) and 6, 8, and 10 weeks after the last laser application in respective eyes. ANX776, provided by Novai (Reading, UK), was diluted with physiological saline solution and intravenously administered at 0.02 mg/kg to each animal. Blood collection and retinal imaging were performed thereafter. SLO imaging was performed to detect DARC signals in the retina using a confocal scanning laser ophthalmoscope (cSLO; Spectralis HRA; Heidelberg Engineering GmbH, Heidelberg, Germany) at regular intervals (pre-dose and 120 and 240 minutes after ANX776 administration). Images were acquired using the ICGA settings on the cSLO^10^ using high resolution, “manual” brightness control, “1 second” cyclic buffer size, “internal” default fixation target, and IR single button with a 30° field of view. Laser intensity was gradually reduced from 100% to optimize the image and ensure it was not oversaturated. Focusing was directed at the RNFL, which was visualized by distinctive striations in IR imaging mode. Once this was achieved, “Automatic Real-time Tracking” was activated, and 100 frames of normalized images were captured. Individual SLO images at pre-dose were used as negative controls to eliminate nonspecific autofluorescence on the corresponding SLO images at 120 and 240 minutes. As in DARC clinical studies, the DARC count was defined as the number of ANX776-positive spots seen in each DARC retinal image after baseline spot subtraction.^9^ SLO imaging was performed on anesthetized animals with pupils dilated, and only the central 30° field of view was captured and analyzed, as previously described.^10^ DARC cells in each image were identified using Novai's convolutional neural network (CNN) (see reference 10 for details). As reported,^10^ the CNN, trained on human healthy eyes, performed well on unseen human eyes with Glaucoma (85.7% sensitivity and 91.7% specificity). Although human and our nonhuman primates share similar RGC size,^12^^,^^13^ the CNN has not been previously exposed to nonhuman primates, a manual verification step was included to finalize the DARC counts for further analysis.

### Spectral Domain Optical Coherence Tomography Imaging

After ocular instillation of a mydriatic (Mydrin-P), animals were anesthetized by intramuscular injection (0.2 mL/kg, 10 mg/kg) of ketamine. Spectral domain optical coherence tomography (SD-OCT) imaging was then performed using a Spectralis OCT device (Heidelberg Engineering GmbH, Heidelberg, Germany). In eyes that received a single laser application, SD-OCT imaging was conducted before (pre = 0) and 5, 7, and 9 weeks after the first laser application. In eyes that received a double laser application, SD-OCT imaging was conducted before the first laser application (pre = 0) and 3, 5, 7, and 9 weeks after the second laser application. The ONHs in both eyes were circularly scanned, and peripapillary sector (temporal superior, temporal, temporal inferior, nasal inferior, nasal, and nasal superior) and global RNFLTs were measured.

### Annexin V Measurement and Pharmacokinetic Evaluation of ANX776

PK of ANX776 was assessed in four healthy male cynomolgus monkeys (body weight, 5.38–5.84 kg; age, 5 years at initiation of the study). A single injection of either 0.0067 mg/kg or 0.02 mg/kg of ANX776 (two animals per group) was administered intravenously to each animal at SNBL. Blood samples were taken from the femoral vein pre-dose and at 5, 15, 30, 60, 120, and 300 minutes after administration of ANX776. For PK assessment of ANX776 in OHT monkeys, blood samples were taken from the femoral vein of four monkeys 120 minutes after ANX776 administration. Serum samples from OHT monkeys and healthy monkeys were frozen at −80°C until measurement of annexin V. Concentrations of ANX776 in sera were estimated using a commercial enzyme-linked immunosorbent assay kit (Abcam, Cambridge, UK) according to the manufacturer's instructions. For PK assessments of ANX776 in healthy monkeys, pharmacokinetic parameters, namely maximum serum concentration of annexin V (C_max_), time of maximum serum concentration (T_max_), area under the concentration-time curve from 0 to 300 minutes (AUC_0__–__300 min_) and half-life of serum annexin V (T_1/2_), were calculated from individual data using the noncompartmental analysis model in Phoenix WinNonlin ver.8.1 (Certara, Princeton, NJ, USA) and Microsoft Excel 2016 (Microsoft Corporation, Redmond, WA, USA). Data are presented as the average of two animals in each group.

### Statistical Analysis

Data are expressed as mean ± standard deviation. DARC count was calculated as a percent of the total counts obtained from time 0 to 10 weeks after the last laser application in each animal. Differences in IOP and the DARC count were statistically analyzed using Dunnett's multiple comparisons test following one-way analysis of variance. Global RNFLT was statistically compared using Dunnett's multiple comparisons test after mixed effects analysis. Pearson's correlation coefficient (one-tailed) was calculated to evaluate the relationship between cumulative DARC counts and reductions in RNFLT in laser-treated eyes. Cumulative DARC counts were calculated as the area under the curve of DARC counts from time 0 to each relevant time point. *P* < 0.05 was used to indicate significance. All statistical analyses were performed using GraphPad Prism 8.0.2 (Graph Pad Software, San Diego, CA, USA).

## Results

### Development of a Laser-Induced NHP OHT Model

To evaluate the DARC signal in the retina under elevated IOP conditions, we investigated an NHP OHT model by irradiating both eyes of cynomolgus monkeys with lasers, as previously described.^5^^,^^6^ Baseline demographics of the animals used for the OHT model are summarized in the Table.

**Table.** Baseline Demographics of Animals Used for the OHT Model

Feature	Glaucomatous Eyes^*^
N	7/8^†^
Age (y)	2^‡^
Sex	Male
IOP (mm Hg)	15.7 ± 1.4
OCT parameter (µM)	
Nasal superior RNFLT	111.71 ± 22.83
Nasal RNFLT	58.29 ± 4.64
Nasal inferior RNFLT	121.00 ± 26.20
Temporal inferior RNFLT	168.14 ± 27.21
Temporal RNFLT	77.57 ± 9.41
Temporal superior RNFLT	146.86 ± 17.49
Global RNFLT	102.29 ± 13.55

* Values are mean ± standard deviation unless otherwise indicated.

† One eye was excluded from analysis.

‡ Age at initiation of the study.

**Figure 1.**
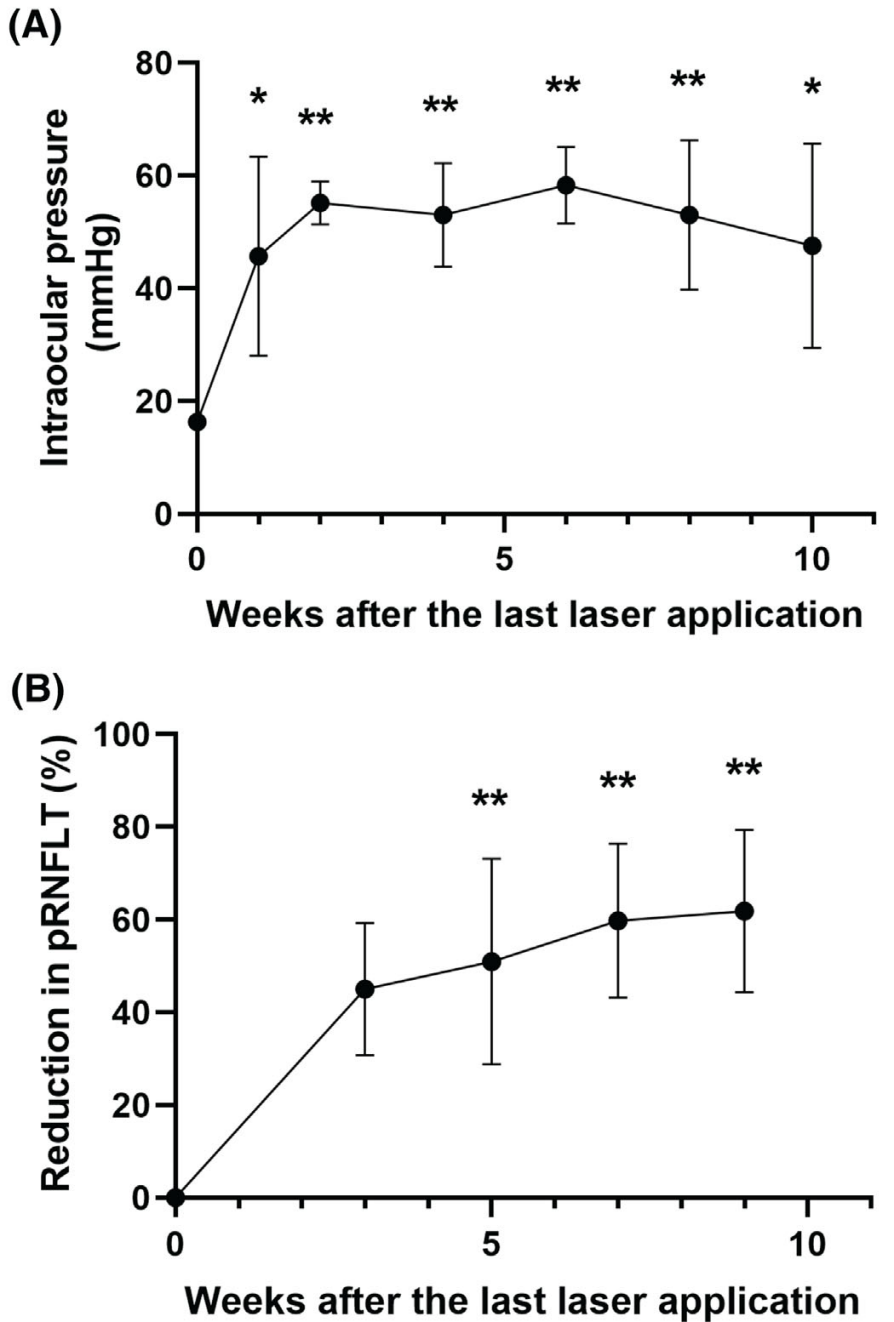
Effect of laser application-induced IOP elevation on global RNFLT. (**A**) IOP elevation was sustained after the last laser application. Dunnett's multiple comparisons test showed that the IOP at each time point after the last laser application was significantly higher than that at baseline. (**B**) Thinning of global RNFLT after laser application. The reduction in global RNFLT was calculated as a percent of the global RNFLT at baseline. Dunnett's multiple comparisons test showed that the global RNFLT at 5, 7, and 9 weeks after the last laser application was significantly reduced compared to that at baseline. *P < 0.05, **P < 0.01. pRNFLT, peripapillary retinal nerve fiber layer thickness. Values are mean ± standard deviation.

The IOP in four eyes became elevated, defined as 30 mm Hg or higher, after the first laser application. In contrast, the IOP in only three eyes met this criterion after the second laser application conducted 14 days after the first laser application. After the laser application, IOP remained significantly elevated above baseline in the eyes of OHT monkeys for at least 10 weeks (Fig. 1A; baseline: 16.3 ± 1.5 mm Hg; 1 week after laser: 45.7 ± 17.7 mm Hg, *P* = 0.02; 2 weeks after laser: 55.1 ± 3.8 mm Hg, *P* < 0.01; 4 weeks after laser: 53.0 ± 9.2 mm Hg, *P* < 0.01; 6 weeks after laser: 58.3 ± 6.8 mm Hg, *P* < 0.01; 8 weeks after laser: 53.0 ± 13.3 mm Hg, *P* < 0.01; 10 weeks after laser: 47.6 ± 18.1 mm Hg, *P* = 0.01). The left eye of animal no. 1 was excluded from analysis because a high IOP could not be established after the first laser application and a second laser application could not be performed because of opacity around the trabecular meshwork.

Laser application also led to a significant reduction in global RNFLT compared to baseline (Fig. 1B; baseline: 0%; 5 weeks after laser: 50.92% ± 22.19%; 7 weeks after laser: 59.78% ± 16.60%; 9 weeks after laser: 61.85% ± 17.51%; *P* < 0.01). Significant thinning of the peripapillary RNFLT in each sector was also observed (Supplementary Fig. S1), suggesting progressive RGC degeneration secondary to IOP elevation. Additionally, the reduction in RNFLT both in the peripapillary sectors and globally were significantly associated with the cumulative IOP elevation (Supplementary Fig. S2). In this study, an OCT image from the right eye of animal no. 1 at 9 weeks after the last laser application could not be obtained because of opacities of the cornea and anterior chamber.

### Detection of OHT-Induced Retinal Cell Apoptosis by DARC

**Figure 2.**
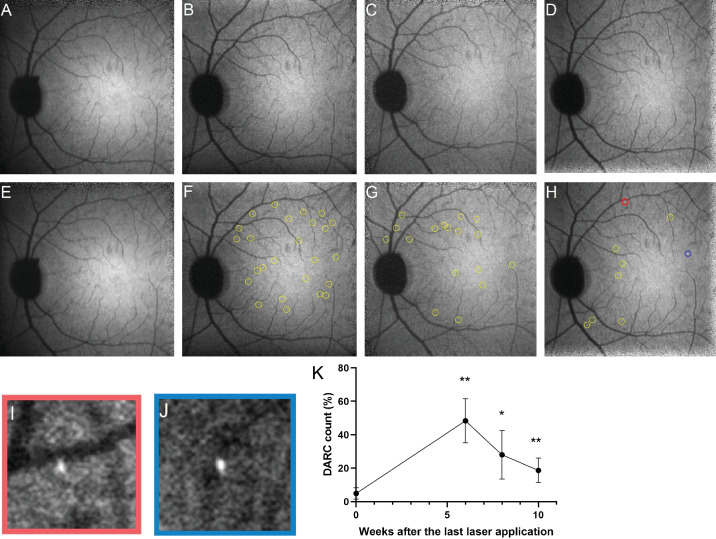
DARC counts increased in the retina of the NHP OHT model. (**A**–**J**) Representative retinal images from OHT monkeys taken 120 minutes after intravenous injection of 0.02 mg/kg of ANX776. Panels show ANX776-positive spots unmarked (**A**–**D**) and marked (**E**-**H**) with *yellow*, *red*, and *blue rings*. (**A**, **E**) Retinal images taken before laser application. (**B**, **F**) Retinal images taken 6 weeks after the last laser application. (**C**, **G**) Retinal images taken 8 weeks after the last laser application. (**D**, **H**) Retinal images taken 10 weeks after the last laser application. (**I**, **J**) Higher magnification images of DARC signals with rings of corresponding colors in image **H**. Changes in DARC counts over time after the last laser application. (**K**) DARC count was calculated as a percent of the total counts obtained from time 0 to 10 weeks after the last laser application. Dunnett's multiple comparisons test showed that DARC count at 6, 8, and 10 weeks after laser application were significantly higher than those at baseline. *P < 0.05, **P < 0.01. Values are mean ± standard deviation.

ANX776-positive cells were visualized as hyperfluorescent DARC spots using fundus fluorescent imaging in the eyes of OHT model monkeys. By focusing on nerve fiber bundles in IR reflection mode in the cSLO, we assumed that DARC spots originated from the RGC layer. Figure 2A–H shows the typical change in appearance of DARC spots over time in the NHP OHT model 120 minutes after intravenous injection of 0.02 mg/kg of ANX776. A relative count of DARC, calculated as a percent of the total spots obtained from time 0 to 10 weeks, was significantly increased in lasered eyes, and the peak increase was seen 6 weeks after the last laser application (Fig. 2K; mean DARC count ± standard deviation; baseline: 4.9% ± 3.3%; 6 weeks after laser: 48.4% ± 13.2%; 8 weeks after laser: 28.0% ± 14.5%; 10 weeks after laser: 18.7% ± 7.4%). DARC count at each point from 6 weeks to 10 weeks also significantly differed from that at week 0 (mean difference from baseline and 95% confidence interval of the difference; 6 weeks after laser: 43, 27–60, *P* < 0.01; 8 weeks after laser: 23, 4.2–42, *P* = 0.02; and 10 weeks after laser: 14, 6.2–21, *P* < 0.01. None of the values was below the baseline value.). These results suggest that DARC technology can be used to detect apoptotic cells in living eyes in an NHP OHT model to gain insight into the degree of damage to RGCs at a particular time point. The relative increase in DARC counts in images taken 120 minutes after ANX776 administration and the pattern of the increase over time after the last laser application were similar to those using images taken at 240 minutes, whereas the number of DARC counts at 120 min was obviously higher than that at 240 minutes (Supplementary Figs. S3A, S3B). In this study, data of DARC counts at 240 minutes in the right eye of animal no. 3 at 8 weeks could not obtained because of the poor quality of SLO imaging. Furthermore, DARC-positive cell counting of the left eye of animal no. 3 at 240 minutes after ANX776 administration at 10 weeks could not be performed because of corneal opacity.

### Correlations Between DARC Count and RNFLT in Laser-Treated Eyes

**Figure 3.**
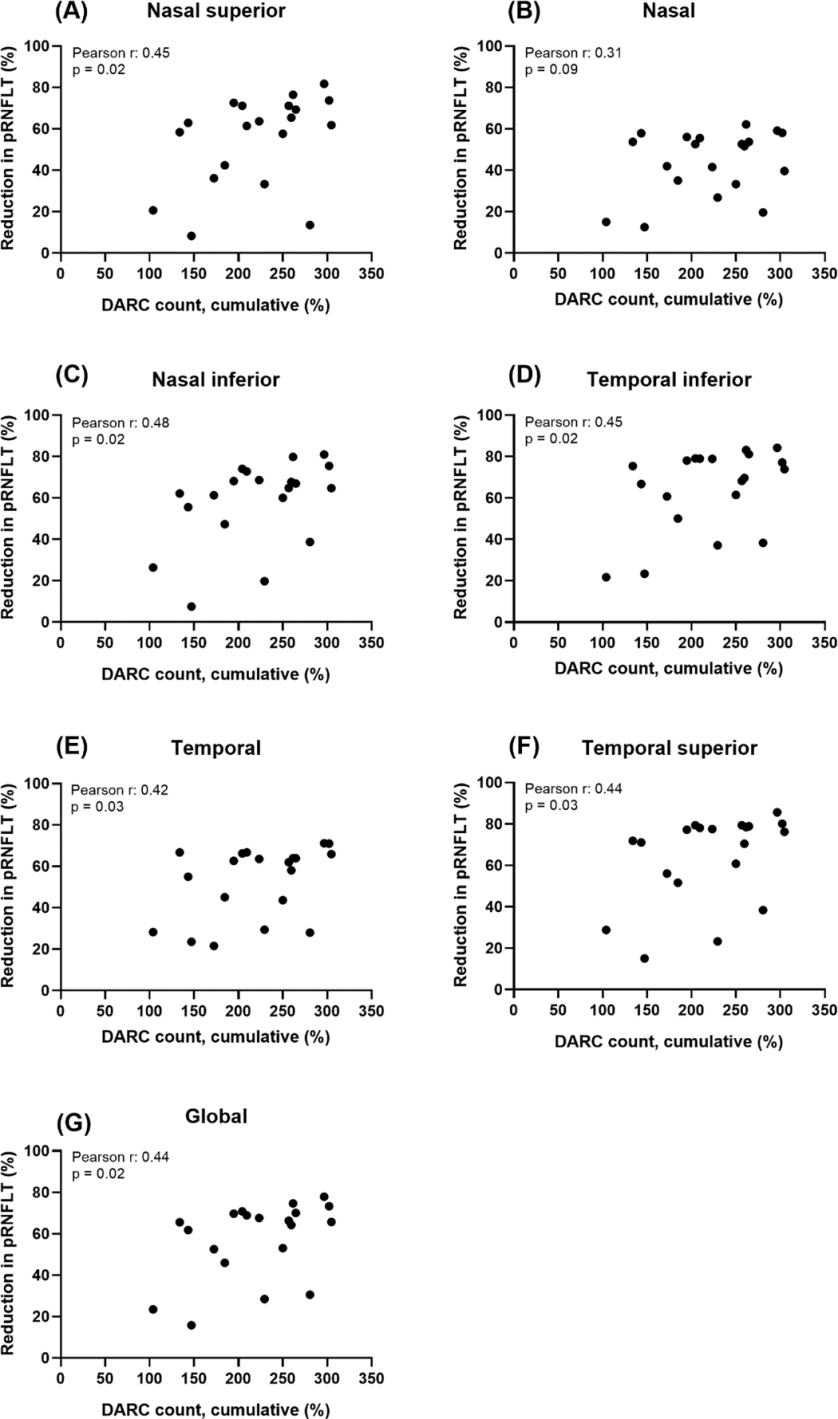
Relationship between cumulative DARC counts and reduction in RNFLT in laser-treated eyes. (**A**–**G**) Cumulative DARC counts were calculated as the area under the curve of DARC counts from time 0 to each relevant time point. Time points are expressed as weeks after the last laser application. We matched DARC counts obtained at weeks 6, 8, and 10 to RNFLTs measured at weeks 5, 7, and 9 after the last laser application, respectively, because DARC counts and RNFL measurements were performed within a week of each other. Using Pearson's correlation coefficients (one-tailed), a significant correlation between cumulative DARC counts and reduction in thickness was noted for the nasal superior (*r* = 0.45, *P* = 0.02), nasal inferior (*r* = 0.48, *P* = 0.02), temporal inferior (*r* = 0.45, *P* = 0.02), temporal (*r* = 0.42, *P* = 0.03), and temporal superior (*r* = 0.44, *P* = 0.03) sectors and global RNFLT (*r* = 0.44, *P* = 0.02). pRNFLT, peripapillary retinal nerve fiber layer thickness; DARC, detection of apoptosing retinal cells.

Pearson's correlation coefficients (one-tailed) were calculated to evaluate the relationship between the cumulative DARC count and reduction in RNFLT in each peripapillary sector or global RNFLT. Cumulative DARC count was calculated as the area under the curve of DARC counts from time 0 to the relevant time point. We matched DARC counts obtained at weeks 6, 8, and 10 to RNFLTs measured at weeks 5, 7, and 9 after the last laser application, respectively, because although DARC counts and RNFLT measurements were performed at slightly different time points, they were within a week of each other. Figure 3 shows that there were significant correlations between the cumulative DARC count and the reduction in RNFLT in the nasal superior (*r* = 0.45, *P* = 0.02), nasal inferior (*r* = 0.48, *P* = 0.02), temporal inferior (*r* = 0. 45, *P* = 0.02), temporal (*r* = 0.42, *P* = 0.03), and temporal superior (*r* = 0.44, *P* = 0.03) sectors and global RNFLT (*r* = 0.44, *P* = 0.02).

### Pharmacokinetics of ANX776

**Figure 4.**
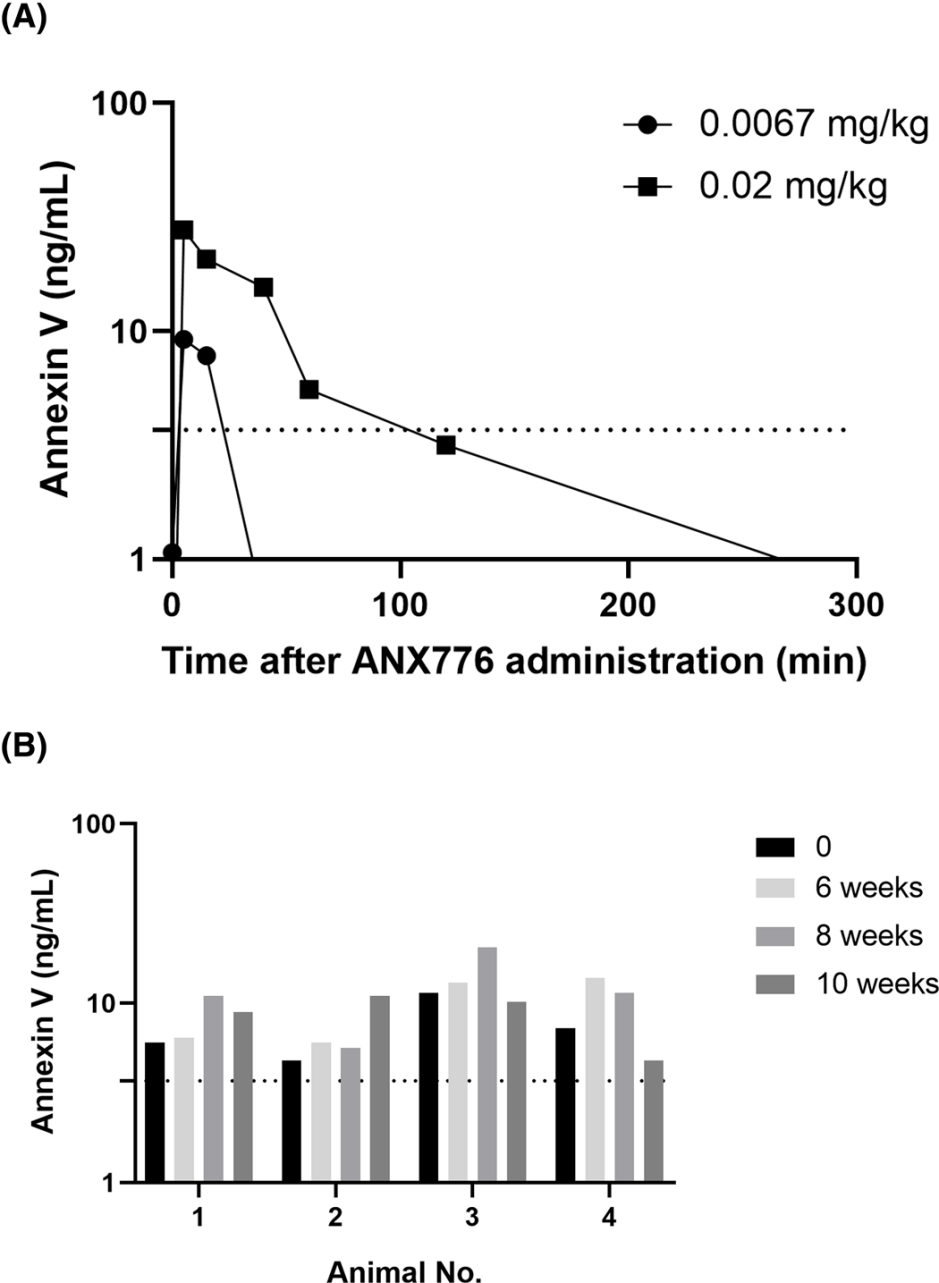
Annexin V concentrations in blood after ANX776 administration of untreated healthy monkeys and OHT model monkeys that received laser application. (**A**) Mean serum concentration of annexin V over time after single intravenous administration of ANX776 at two different doses. Annexin V concentration was below the limit of quantification at 40 minutes and 120 minutes after administration of 0.0067 mg/kg and 0.02 mg/kg ANX776, respectively. (**B**) Mean serum concentration of annexin V 2 hours after ANX776 administration at each DARC-measurement time point (weeks after the last laser application). Animal no. 3 was considered to have received double laser application, even though its right eye was lasered once, whereas its left eye was lasered twice; data for this animal are expressed in terms of weeks after the second laser application. *Dotted line* in each graph shows the limit of quantification (3.7 ng/mL).

We studied the PK of ANX776 after single intravenous administration to monkeys that had not received laser application. ANX776 in blood showed dose-dependent exposure (Fig. 4A). Mean C_max_, mean T_max_, mean AUC_0-300 min_, and mean T_1/2_ of 0.02 mg/kg ANX776 was 27.8 ng/mL, 5.00 min, 983 min*ng/mL, and 24.5 min, respectively. Serum levels of ANX776 after treatment with 0.02 mg/kg of ANX776 were similar to those observed in humans at the optimal dose (0.4 mg/subject).^8^ In contrast, on multiple repeated dosing of ANX776 to animals that had received laser application, levels of ANX776 in serum remained steady 2 hours after dosing, and no rapid decline was observed during the study period (Fig. 4B).

## Discussion

By examining and characterizing DARC counts in the retinas of OHT monkeys, we have derived a set of basic data that can be used to design pharmacodynamics study protocols to investigate neuroprotective agents. This experimental NHP model of OHT partly mimics the pathology of clinical glaucoma^11^ in terms of RNFLT thinning around ONH.

Glaucomatous damage, associated with RGC degeneration via apoptosis, persists in almost 50% of patients, despite sufficient IOP control,^14^ suggesting that there is a high unmet medical need for novel therapeutics for glaucoma that can prevent or delay glaucomatous neurodegeneration independently of IOP. However, because glaucomatous optic neuropathy is a slowly progressive disease, clinical trials on neuroprotection are necessarily long in duration.^15^ Thus estimating the neuroprotective potential of candidate drugs in preclinical/clinical studies with short study durations and small sample sizes is incredibly difficult. Determining the neuroprotective activity of a candidate in preclinical/clinical settings would require an analytical method with high reliability and specificity/sensitivity to enable detection of small signs of efficacy. Given that the degree of apoptosis in the retina may predict the rate of RGC loss, a major hallmark of glaucoma, apoptosis may be a potential biomarker of glaucoma that can be used to evaluate how well a patient is responding to a given treatment.

DARC is a noninvasive real-time imaging technique that uses cSLO to visualize apoptosis of single retinal nerve cells in vivo via intravenous administration of ANX776. Accumulating evidence suggests that DARC technology could be a promising biomarker platform for early detection of disease activity that leads to glaucomatous damage. Clinical trials that used DARC technology in glaucoma patients have shown that intravenous ANX776 is safe and well tolerated and that DARC, analyzed using an artificial intelligence (AI)-aided algorithm, can be used to predict disease activity.^7^^,^^10^ The algorithm was reported to have 97.0% accuracy, 91.1% sensitivity, and 97.1% specificity for spot detection in human eyes.^10^ Although the objective of our present study was not to validate this AI model in primates, our results are indicative of accurate DARC spot detection in primates, albeit that further studies are needed to confirm these findings.

Here, we examined DARC in a laser-induced NHP OHT model. Although several preclinical studies have used DARC to assess disease and treatment, we are to our knowledge the first to use DARC in an NHP experimental model of OHT.^16^^–^^21^ The retinas of NHPs have many anatomical similarities to those of humans.^22^^,^^23^ For example, NHPs have a fovea, which is absent in dogs, pigs, and rodents and are accordingly important for preclinical research on macular diseases. Moreover, the laser-induced NHP OHT model at least partly mimics the impaired trabecular outflow observed in clinical glaucoma in humans that contributes to the elevated IOP in POAG.^24^ Previous reports have described the use of intravitreally injected Alexa Fluor 488-labeled Annexin 5 for imaging detection of apoptotic RGCs in monkeys. Induction in this model was by intravitreal injection of staurosporine, however, which has a markedly severe effect, with rapid and extensive apoptotic RGCs observed as early as 1 hour after injection.^16^

Similar to previous findings, our application of a laser to the trabecular meshwork of the eyes of monkeys led to sustained IOP elevation accompanied by significant reductions in RNFLT globally and in each peripapillary sector.^11^^,^^25^ Of the various OCT parameters used to evaluate glaucoma, thinning of the RNFLT around the ONH is perhaps the most commonly used for diagnosing and monitoring glaucoma.^26^ The RNFL consists primarily of RGC axons, which progressively degenerate in glaucoma, resulting in the thinning and disappearance of axon bundles from the RNFL.^27^ Furthermore, laser irradiation also increased DARC-positive apoptotic retinal cells, as detected using ANX776. The increase in DARC counts was more evident when cSLO imaging was performed at 120 minutes after ANX776 administration than at 240 minutes, which is consistent with a clinical DARC study that used images taken at 120 minutes after ANX776 administration for analysis. Moreover, our finding that the administered dose of ANX776 (0.02 mg/kg) demonstrated a comparable PK profile, including mean C_max_, mean T_max_, mean AUC_0-300min_ and mean T_1/2_, to the optimal dose used in humans (0.4 mg/subject)^7^ indicates that this preclinical analysis used similar experimental conditions to those in clinical DARC analysis. In the future, however, we believe it will be important to adopt experimental designs that facilitate comparisons between independent groups and contrasts between different experimental interventions.

In the correlation analysis, cumulative DARC counts were significantly associated with the reduction in RNFLT both in peripapillary sectors and globally. Indeed, comparing the change in DARC count and reduction in RNFLT, it was suggested that a high DARC count is associated with a high rate of RNFL thinning (around 5 to 6 weeks after laser application). As the rate of DARC count decreased, so did the rate of RNFL thinning (around 7 to 8 and 9 to 10 weeks after last laser application). Bearing in mind the clinical findings that DARC levels predict future progression,^4^^,^^10^ it would have been more appropriate for DARC readouts to have been obtained earlier—perhaps at weeks 2 and 4, to better examine the predictive capabilities on DARC, and this should be included in future work. Cumulative DARC counts determined using cSLO are indicative of the accumulation of apoptotic cells, mostly RGCs, in the retina of OHT model monkeys because cSLO for DARC counting is focused to visualize axon bundles from the RNFL and cells in the RGC layer. Taken together, these findings suggest that DARC counts in the retina could be a potential biomarker for monitoring RGC apoptosis in the NHP OHT model and in clinical glaucoma.

In this study, one eye was excluded from the analysis because an additionally required laser application could not be performed because of opacity. Furthermore, some SLO and OCT images of tested eyes could not be obtained because of opacity on the day of measurement and were excluded from the analysis. Opacity could well impact laser irradiation, imaging, and the ability to detect the DARC signal and create variability in the data. Controlling the appearance of opacity in tested eyes is an important issue for the future.

Our study has some limitations. First, we examined relatively few monkeys and did not test any neuroprotective agents. Further studies with sufficient animal numbers are needed to optimize a protocol that uses DARC technology in an NHP OHT model to evaluate the pharmacodynamic response to neuroprotective agents. We would particularly welcome a protocol that assesses the effect of therapy on DARC activity and its correlation with future RNFLT changes. Second, the study lacked a control group or control eye. This constrains the extent of the inferences that can be drawn. Given financial and ethical costs to preclinical trials, future studies of similar numbers of subjects could be improved by including a typical comparison of both eyes separated into a control/sham or experimental condition. Third, laser application was independently applied to the left and right eyes of each monkey for high IOP induction, and the influence of the left and right eyes on each other was not considered. Fourth, animals that received single and double laser application were combined in the study; thus we did not consider any differences between these animals.

In conclusion, our data indicate that the laser-induced OHT cynomolgus monkey model, which shows increased DARC counts, sustained IOP elevation and reduced peripapillary RNFLT, mimics the pathophysiology of clinical glaucoma. Cumulative DARC counts are significantly associated with thinning of the peripapillary RNFL, which consists primarily of RGC axons. These findings suggest that DARC technology can effectively assess glaucomatous optic nerve damage in a NHP model of OHT. Additional studies with larger sample sizes are needed to further optimize the design of pharmacodynamic studies using DARC technology in the NHP OHT model.

## Supplementary Material

Supplement 1
