# Supplementary material for: Real-Time Imaging of Single Retinal Cell Apoptosis in a Non-Human Primate Ocular Hypertension Model
Source: Transl Vis Sci Technol. 2024 Jan 22;13(1):20. doi: 10.1167/tvst.13.1.20 (PMC10810027; doi:10.1167/tvst.13.1.20)
Supplement: Supplement 1 [file tvst-13-1-20_s001.pdf]

Supplementary Figure S1

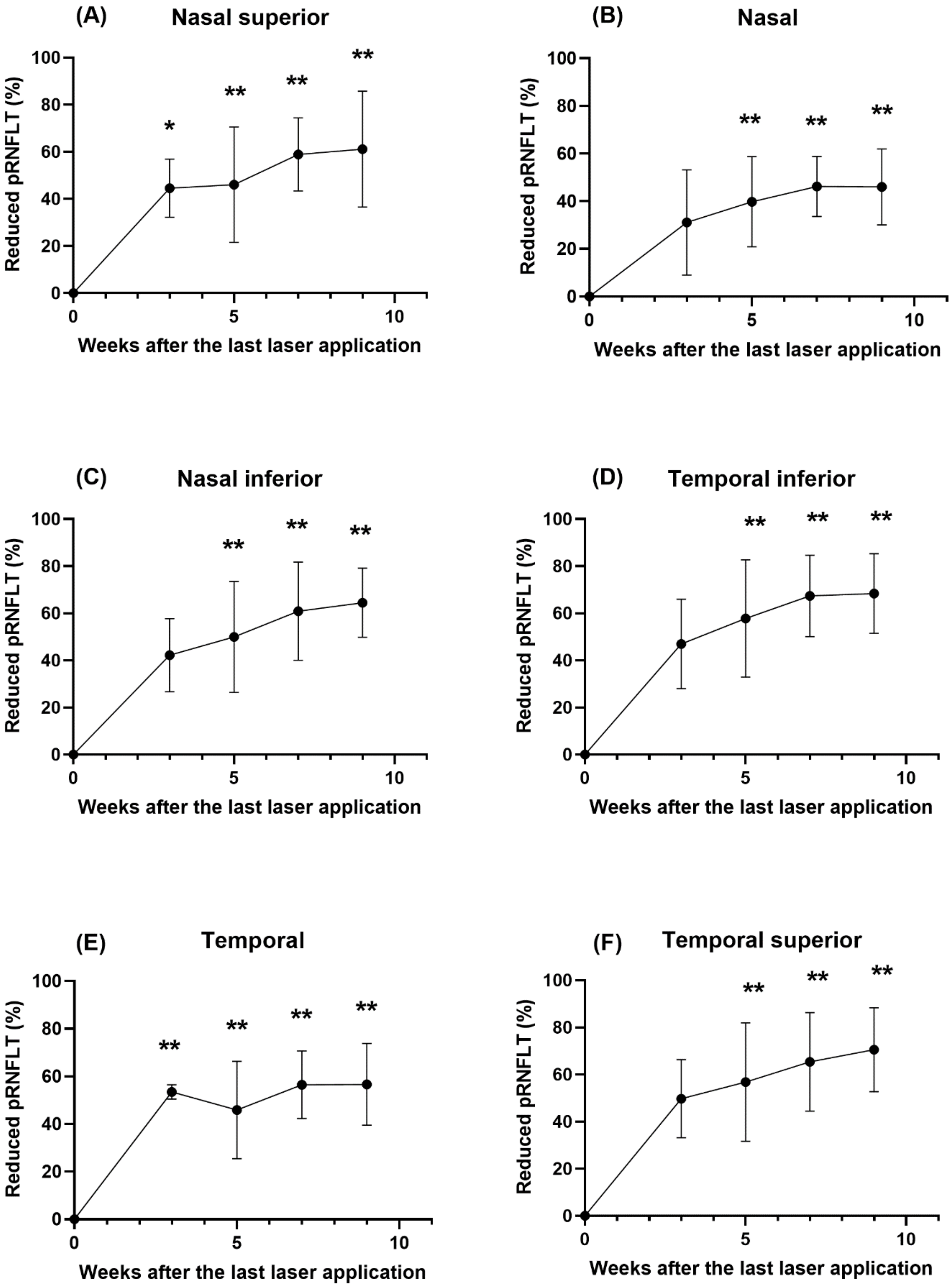

Supplementary Figure S1 Effect of laser application-induced IOP elevation on peripapillary sector RNFLT. Laser application reduced the peripapillary RNFLT in all sectors. The reduction in peripapillary RNFLT in each sector was calculated as a percent of the RNFLT at baseline. Dunnett's multiple comparisons test showed that the RNFLT in each peripapillary sector at each time point after the last laser application was significantly reduced compared to baseline. $*p < 0.05$, $**p < 0.01$. pRNFLT, peripapillary retinal nerve fiber layer thickness. Values are mean ± standard deviation.

Supplementary Figure S2

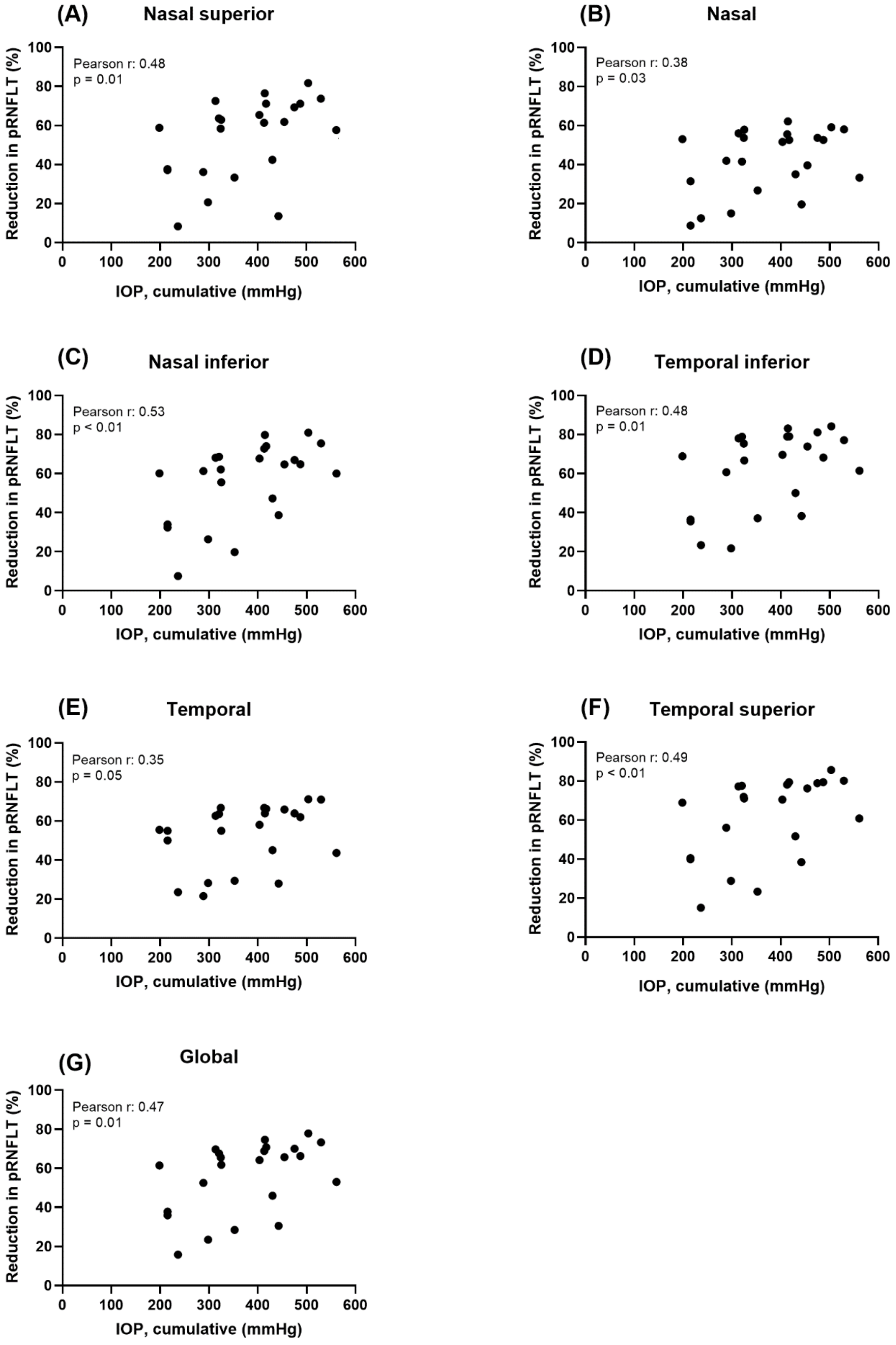

Supplementary Figure S2 Relationship between cumulative IOP elevation and reduction in RNFLT in laser-treated eyes. Pearson's correlation coefficients (one-tailed) were calculated to evaluate the relationship between cumulative IOP elevation and reduction in RNFLT in laser-treated eyes. (A-G) Cumulative IOP elevation was calculated as the area under the curve of IOP from time 0 to the relevant time point. Time points indicate weeks after the last laser application. We matched the IOP elevation measured at week 4, 6, 8, and 10 to the RNFLT measured at week 3, 5, 7, and 9 after the last laser application, respectively, because IOP and RNFL measurements were performed within a week of each other. A significant correlation was noted between cumulative IOP elevation and a reduction in thickness in the nasal superior ($r = 0.48$, $p = 0.01$), nasal ($r = 0.38$, $p = 0.03$), nasal inferior ($r = 0.53$, $p < 0.01$), temporal inferior ($r = 0. 48$, $p = 0.01$), temporal ($r = 0.35$, $p = 0.05$), and temporal superior ($r = 0.49$, $p < 0.01$) sectors and global RNFLT ($r = 0.47$, $p = 0.01$). pRNFLT, peripapillary retinal nerve fiber layer thickness; IOP, intraocular pressure.

Supplementary Figure S3
(A)

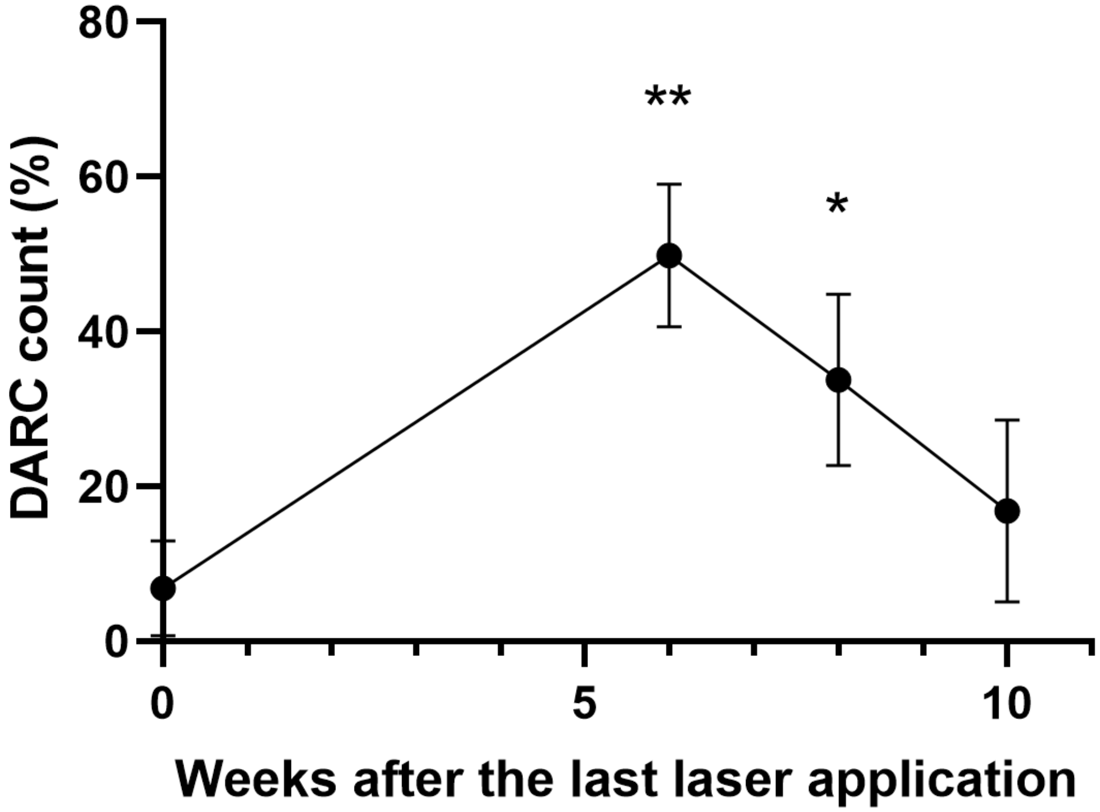

(B)

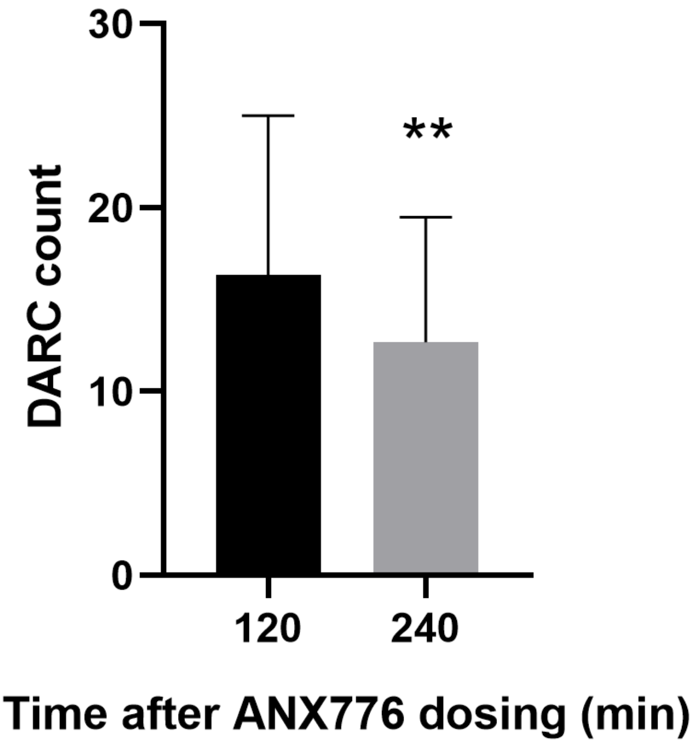

Supplementary Figure S3 DARC counts increased in the retina of the NHP OHT model in SLO images taken 240 min after ANX776 administration. (A) Change in DARC count over time after the last laser application. SLO images for DARC counting were obtained 240 minutes after intravenous injection of 0.02 mg/kg of ANX776.

DARC count was calculated as a percent of the total counts obtained from time 0 to 10 weeks after the last laser application in each animal. Dunnett's multiple comparisons test showed that relative DARC counts at 6 and 8 weeks after laser application were significantly higher than that at baseline. $*p < 0.05$, $**p<0.01$. (B) Number of 120 min-DARC counts obtained from week 6 to week 10 was compared with that of 240 min-counts. Paired t test showed that DARC counts at 120 min were significantly higher than those at 240 min. $**p < 0.01$. DARC counts at 240 min in the right eye of Animal No. 3 at 8 weeks is missing due to the poor quality of SLO imaging, and 240 min-DARC counts in the

left eye of Animal No. 3 at 10 weeks are also missing due to corneal opacity. Corresponding data pairs at 120 min and 240 min were also excluded from statistical analysis. Values are mean ± standard deviation.
